# Improvements in Extraction Methods of High-molecular-weight DNA from Soils by Modifying Cell Lysis Conditions and Reducing Adsorption of DNA onto Soil Particles

**DOI:** 10.1264/jsme2.ME21017

**Published:** 2021-07-06

**Authors:** Yoriko Sakai

**Affiliations:** 1 Institute for Agro-Environmental Sciences, National Agriculture and Food Research Organization (NARO), 3–1–3, Kannondai, Tsukuba, Ibaraki, Japan

**Keywords:** soil DNA extraction, high-molecular-weight DNA, mechanical treatment, lysozyme, Andosols, sonicated and boiled DNA

## Abstract

High-molecular-weight DNA (HMW DNA) extracted from soil is useful for examined the functions and diversity of soil organisms, the majority of which are difficult to culture. In the present study, the procedures used to extract HMW DNA from soil samples were improved. The grinding of soil samples with liquid nitrogen followed by a lysozyme treatment at 45°C for 1 h and an incubation with protease and SDS at 50°C for 5 h increased the size and yield of HMW DNA extracted from these samples. In the soil group Andosols, the addition of boiled sonicated salmon DNA was effective for HMW DNA extraction.

Due to the difficulties associated with culturing the majority of soil organisms, their DNA is directly extracted from soil to examine their distribution and functions in the environment (Quince *et al.*, 2017). This DNA is also used to obtain useful genes from soil. High-molecular-weight DNA (HMW DNA) is also useful for procuring clustered functional genes in soil organisms (Lam *et al.*, 2015). Some of the gene clusters used in the biosynthesis of antibiotics are larger than 100‍ ‍kb (Sinha *et al.*, 2019). Williamson *et al.* (2005) successfully cloned DNA fragments, measuring up to 190‍ ‍kb, isolated from soil samples into bacterial artificial chromosome (BAC) vectors. Several libraries of HMW DNA isolated from soil have been constructed, and various functional genes have been analyzed (Brady and Clardy, 2000; Rondon *et al.*, 2000; MacNeil *et al.*, 2001; Quaiser *et al.*, 2002; O’Mahony *et al.*, 2015; Bouhajja *et al.*, 2017; Duarte *et al.*, 2017). Clone libraries enable the expression and analysis of target genes and the use of some products from gene clusters. A recent study reported that due to advances in sequence technology, long-read sequencing is advantageous for genome analyses (Jain *et al.*, 2018). This technology has also been applied to the extraction of DNA from environmental samples (Johnson *et al.*, 2017). Less damaged and biased HMW DNA isolated from environmental samples may also be useful for this technology.

The extraction of HMW DNA from soil samples is performed using two approaches, *i.e.*, direct extraction and indirect extraction. In the indirect extraction method, the microbial fraction is separated from soil particle fractions, and DNA is then extracted from the microbial fraction. Although this method yields a lower amount of DNA than the direct method, the molecular weight of DNA was found to be higher than that extracted using the direct method (Steffan *et al.*, 1988; Gabor *et al.*, 2003). The indirect method is useful for targeting specific microbial groups, such as bacteria and archaea, and for reducing DNA contamination from fungi and larger organisms (Gabor *et al.*, 2003). In contrast, in the direct extraction method of HMW DNA from soil samples, soil materials are directly mixed with a DNA extraction buffer, and the microbial cells present in soil are lysed by enzymes and/or surfactant. The DNA released into the buffer is separated from soil materials and collected. This method targets the microbial cells strongly attached to soil particles, and the DNA yield is markedly higher than that from the indirect method (Steffan *et al.*, 1988). Among the procedures reported for the direct extraction method, that described by Zhou *et al.* (1996) using a high-salt buffer has been applied in several experiments with or without modifications (Brady and Clardy, 2000; Rondon *et al.*, 2000; Williamson *et al.*, 2005; Tao *et al.*, 2011; Bouhajja *et al.*, 2017; Verma *et al.*, 2017). We recognized the efficiency of the method described by Zhou *et al.* (1996) and attempted to identify the most suitable conditions in each step for the extraction of HMW DNA from our soil samples in order to increase yield and molecular weight.

Based on the method of HMW DNA extraction from soil samples described by Zhou *et al.* (1996), we compared three methods: method I (standard method), method II (standard method with grinding), and method III (modified method) (Fig. 1 and Supplementary material), using different soil samples: SA, SB, and SC (Table S1), to identify more effective conditions for the extraction of less damaged HMW DNA from soil. The results of pulsed-field gel electrophoresis (PFGE) of extracted DNA are shown in Fig. 2. The grinding treatment of the three soil samples increased the yield of HMW DNA, and the combination of grinding and modified lysis conditions further increased the yield and size of extracted DNA from the three soil samples. In previous studies, physical treatments, such as the grinding and freeze-thawing of soil samples, were found to be effective for the extraction of HMW DNA from soil organisms, particularly Gram-positive bacteria (Zhou *et al.*, 1996), and these treatments were employed in studies on HMW DNA extraction (Hurt *et al.*, 2001; Williamson *et al.*, 2005; de Castro *et al.*, 2011). Zhou *et al.* (1996) reported the efficiency of a physical treatment on inoculated Gram-positive bacteria. The present study also demonstrated the effectiveness of a physical treatment on the yield and molecular weight of extracted DNA using fresh soil samples. The freeze-thawing treatment step was omitted based on the findings of Moré *et al.* (1994). Lysozyme is frequently used as a cell lysis enzyme to extract DNA from soil samples (Tsai and Olson, 1991; Tebbe and Vahjen, 1993; Frostegård *et al.*, 1999; Brady and Clardy, 2005; Bouhajja *et al.*, 2017; Verma *et al.*, 2017). Although lysozyme generally reacts at 37°C, a temperature of 45°C was selected in our modified procedure based on the results of a pretest (Fig. S1). Lysozyme may react better at a higher temperature in the applied high-salt buffer. The conditions of the incubation with protease and SDS were also changed from the original 2 h at 65°C to 5 h at 50°C in method III. The molecular weight of extracted DNA was slightly larger with the incubation at 50°C than with that at 65°C (data not shown), and the incubation for 5 h yielded the highest ratio of HMW DNA larger than 48‍ ‍kb (Fig. S2). A reduction in the processing time was one of the objectives in the development of the original method by Zhou *et al.* (1996), whereas we prioritized the yield and molecular weight of HMW DNA in our modified procedure.

The yield of HMW DNA from 11 soil samples (Table S1) was compared between the standard method (method I) and modified method (method III) (Fig. 3). To estimate the amount of extractable DNA in each soil sample, the DNA yield obtained using a commercial kit (FastDNA Spin Kit for Soil, MP Biomedicals) with the bead-beating method was also analyzed (details in Supplementary material). The yield of HMW DNA obtained using method III was higher than that using method I in soil samples SA, SB, SC, SD, and SE. However, no significant differences were observed in yields from the other soil samples between methods I and III (*P*<0.05), and yields were significantly lower than those using the bead-beating method without soil sample SG (*P*<0.05). The lower yield of HMW DNA obtained using method III may have been due to the adsorption of HMW DNA onto soil particles, and the addition of skim milk was effective for extracting DNA from these soil samples using the bead-beating method (see Supplementary material).

To reduce the adsorption of HMW DNA onto soil particles, we examined the effects of the addition of skim milk, yeast RNA, or sonicated salmon DNA to soil sample SJ (Fig. 4, detailed methods in Supplementary material). The results obtained showed that the addition of each of the three materials increased the yield of HMW DNA, with the addition of sonicated salmon DNA providing the highest yield. Previous studies using a mechanical treatment without protease reported that the addition of skim milk or casein increased the yield of nucleic acid from some soils (Volossiouk *et al.*, 1995; Hoshino and Matsumoto, 2004; Ikeda *et al.*, 2008; Wang *et al.*, 2012). In the present study, the addition of skim milk affected the extraction of HMW DNA from soil sample SJ; however, efficiency was not as high as expected. This may have been due to the application of protease, the enzymatic activity of which against the soil component may have been decreased by the milk protein as an excess substrate for protease. The addition of RNA has been reported to increase the efficiency of DNA extraction from some soil samples (Frostegård *et al.*, 1999; Hoshino and Matsumoto, 2004; Ikeda *et al.*, 2008). In the present study, the efficiency of the addition of RNA was similar to that of skim milk, but was lower than that of sonicated salmon DNA. This may have been due to RNA being more soluble in the high-salt buffer (pH 8.0) because of the presence of an additional hydroxyl base (Sambrook and Russell, 2001; Watson *et al.*, 2004) as well as lower attachment to soil particles than DNA.

Although the addition of sonicated salmon DNA was effective at yielding HMW DNA, there was a concern regarding the contamination of soil DNA with salmon DNA. HMW salmon DNA was detected in the electrophoresis gel of a sterile soil sample to which salmon DNA was applied (Fig. 4, lane 5). To reduce contamination with salmon DNA, sonicated salmon DNA was denatured and fragmented by boiling for 15‍ ‍min. This treatment reduced the amount of HMW DNA from salmon DNA while maintaining the yield of HMW DNA from the soil sample (Fig. S3). Further harsh treatment reduced the yield. To enhance the adsorption of boiled salmon DNA onto soil particles, salmon DNA was added to soil samples suspended in the extraction buffer, and the mixture was left to stand at a room temperature of approximately 25°C for 1 h before the addition of lysozyme (method IV, Fig. 1). Fig. 3 shows the yield of HMW DNA from soil samples with the addition of boiled sonicated salmon DNA. The addition of boiled sonicated salmon DNA significantly increased the yield of HMW DNA in soil samples SI, SJ, and SK only, which have been classified into Andosols, a volcanic soil group (*P*<0.05). The length of HMW DNA extracted from the three soil samples was analyzed by PFGE (Fig. 5). In soil sample SI, from which only 0.007‍ ‍μg g^–1^ dry soil of HMW DNA was extracted using method III, the addition of salmon DNA increased the yield to 3‍ ‍μg g^–1^ dry soil of HMW DNA.

Although the boiling treatment of salmon DNA reduced contamination, a small amount of salmon DNA may have remained in soil HMW DNA after purification and size selection (Fig. S3). Depending on the purpose of the experiment, the addition of skim milk may be better than salmon DNA to avoid contamination. In most cases, sequence data need to be employed in order to distinguish the nucleotide sequences of soil microbes from those of salmon. In the present study, purification steps were not examined; however, purification methods are necessary when HMW DNA extracted from soil samples is used in molecular biological experiments, such as enzyme restriction, cloning, and PCR. The electrophoresis of crude HMW DNA followed by a gel extraction procedure is effective in most cases. Several purification methods of crude HMW DNA from soil samples have been reported to date (Zhou *et al.*, 1996; Brady and Clardy, 2000; Rondon *et al.*, 2000; MacNeil *et al.*, 2001; Williamson *et al.*, 2005; Kielak *et al.*, 2009; de Castro *et al.*, 2011; O’Mahony *et al.*, 2015; Bouhajja *et al.*, 2017; Verma *et al.*, 2017), and need to be applied depending on the purpose of the experiment. The improved procedures for HMW DNA extraction from soil samples described in the present study and examples of purification steps are shown in Fig. S4.

The compositions of bacterial phyla in HMW DNA (method III) and DNA extracted with the bead-beating method were examined in other soil samples from paddy fields based on the amplicon sequence of the V3–V4 region of 16S rRNA genes (Y. Sakai, unpublished). These compositions were similar each other with some exceptions. Most differences were found in the phylum *Firmicutes*, in which the proportions of the phylum in total bacteria detected in HMW DNA were lower than the quarter of the proportions detected in DNA extracted using the bead-beating method. On the other hand, the proportions of the phyla *Spirochaetes* and *Verrucomicrobia* in total bacteria detected in HMW DNA were up to 1.9-fold higher than the proportions detected in DNA extracted with bead-beating method. Therefore, the method for HMW DNA extraction looks like suitable for microorganisms with soft cell walls and not for robust cells, such as spores, which are formed by many genera of *Firmicutes* (Paul *et al.*, 2019). It might be assumed that HMW DNA originated from more active microorganisms without dormant robust cells, such as spores. Further studies are needed to clarify this hypothesis.

## Citation

Sakai, Y. (2021) Improvements in Extraction Methods of High-molecular-weight DNA from Soils by Modifying Cell Lysis Conditions and Reducing Adsorption of DNA onto Soil Particles. *Microbes Environ ***36**: ME21017.

https://doi.org/10.1264/jsme2.ME21017

## Supplementary Material

Supplementary Material

## Figures and Tables

**Fig. 1.**
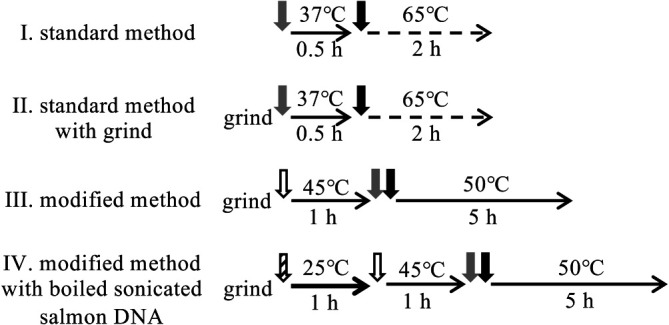
Comparison of 4 methods for extracting HMW DNA from soil samples. Vertical gray, black, white, and striped arrows show the addition of protease, SDS, lysozyme, and sonicated boiled salmon DNA, respectively. Lateral solid, dotted, and bold arrows show a rotating incubation, a standing incubation with gentle inversion every 20‍ ‍min, and a standing incubation, respectively.

**Fig. 2.**
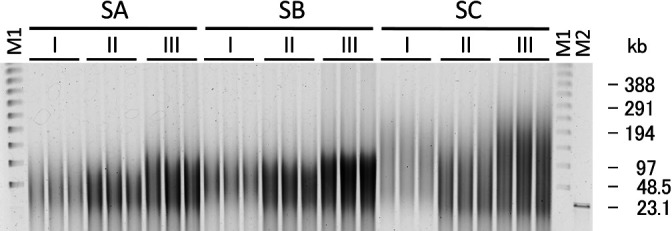
Pulsed-field gel electrophoresis of extracted crude HMW DNA from soil samples SA, SB, and SC using methods I, II, and III. M1: lambda ladder (Bio-Rad); M2: λ/HindIII.

**Fig. 3.**
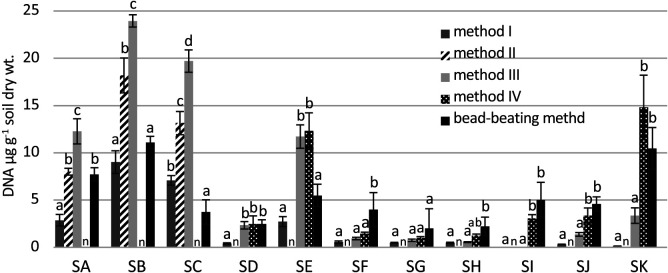
DNA yield from soil samples with several extraction methods. Different letters indicate significant differences within each soil sample (*P*<0.05). n: no data.

**Fig. 4.**
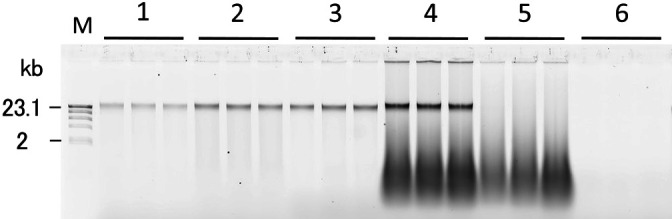
Electrophoresis of crude HMW DNA extracted from soil sample SJ using method I with or without additional material to reduce the adsorption of HMW DNA onto soil particles. Lanes 1–4: nonsterile soil; 5, 6: sterile soil. Lane 1 and 6: no addition; lane 2: skim milk; lane 3: yeast RNA; lanes 4 and 5: sonicated salmon DNA; M: λ/HindIII.

**Fig. 5.**
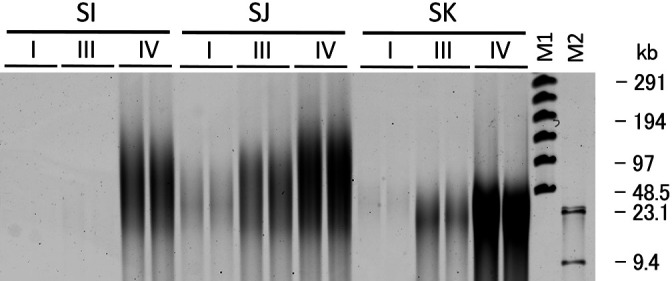
Pulsed-field gel electrophoresis of crude HMW DNA extracted from soil samples SI, SJ, and SK using methods I, III, and IV. M1: lambda ladder (Bio-Rad); M2: λ/HindIII.
