# Supplementary material for: Improvements in Extraction Methods of High-molecular-weight DNA from Soils by Modifying Cell Lysis Conditions and Reducing Adsorption of DNA onto Soil Particles
Source: Microbes Environ. 2021 Jul 6;36(3):ME21017. doi: 10.1264/jsme2.ME21017 (PMC8446751; doi:10.1264/jsme2.ME21017)
Supplement: Supplementary file 1 — Supplementary Material [file 36_21017_s1.pdf]

Supplementary information

Materials and methods

Soil samples

A total of 11 soil samples were used in this study that were collected from agricultural fields (Table S1). The soils of paddy fields were sampled before waterlogging. These soils were sieved with 2-mm mesh and stored at 4°C until use.

Extraction of HMW DNA from soil samples

HMW DNA was extracted from soil samples according to the method reported by Zhou *et al.* (1996), which was applied as a standard method (method I, Fig. 1) with a slight modification. Soil samples weighing 0.4 g and 1 ml of extraction buffer (Zhou *et al.* 1996) were mixed in siliconized 2-ml tubes for 15 s using a vortex mixer. After adding 5 μl of proteinase K solution (Sigma-Aldrich, USA, 20 mg ml^{-1} water), the soil suspensions were incubated at 37°C for 30 min with vertical rotation. Then, 110 μl of 20% SDS was added to the soil suspensions and incubated at 65°C for 2 h with gentle inversions every 20 min. Next, the soil suspensions were centrifuged at 20,000 *g* for 5 min at room temperature, and the supernatants were collected into 1.5-ml tubes, mixed with equal volumes of isopropanol, and incubated at room temperature for 1 h. These samples were centrifuged at 20,000 *g* for 15 min at room temperature, and the resulting pellets of crude DNA were washed with 70% ethanol and suspended in 100 μl of TE buffer. As in method II, the grinding procedure of soil sample was incorporated into method I. The soil sample weighing 0.4 g was ground in liquid nitrogen using a mortar and pestle and then mixed with 1 ml of extraction buffer. In method III, the grounded soil sample as done in method II was mixed with the extraction buffer, and 20 μl of lysozyme solution (500 mg ml^{-1} TE buffer) was added and incubated for 1 h at 45°C with vertical rotation. Next, 5 μl of proteinase K solution (20 mg ml^{-1} water) and 110 μl of 20% SDS were added to the soil suspension, and the mixture was incubated at 50°C for 5 h with vertical rotation. After the incubation, crude DNA was collected as done in method I. Each extraction procedure was conducted in triplicate.

Extraction of soil DNA using bead-beating method

DNA extraction using the bead-beating method was performed using a FastDNA Spin Kit for Soil (MP Biomedicals, USA) according to the manufacturer’s instructions. Skim milk (BD Difco, USA) was added (Hoshino and Matsumoto 2004) depending on the result of the pretest (data not shown). To 0.4 g of each of the soil samples SF, SG, SH, SI, SJ, and SK, 40, 80, 80, 160, 120, and 120 μl of 20% skim milk solution (in water) was added, respectively.

Analysis of DNA extracted from soil samples

To compare the amount and length of the extracted HMW DNA, the crude DNA was loaded on 1% agarose gel of PFGE. The electrophoresis was performed in a 0.5× TBE buffer using a CHEF-DRII apparatus (Bio-Rad, USA) at 0.6 V mm^{-1}, with a switch time ramping from 90 to 150 s per pulse at 14°C for 16 h. The gel was stained with EtBr and then scanned with an imaging system, Pharos FX (Bio-Rad, USA). To estimate the amount of HMW DNA, the crude DNA was loaded on 0.7% agarose gel, and the electrophoresis was performed in a 0.5× TBE buffer for 30 min at 9.0 V mm^{-1}. The gel was stained with EtBr and then scanned, and the signal intensity of the DNA band larger than 23 kb was analyzed as HMW DNA using the imaging system. To generate the standard curve for estimating the amount of HMW DNA, 50 and 20 ng of lambda DNA (Nippon Gene, Japan) was loaded on the same gel. Statistical analysis of the DNA amounts in each soil sample were subjected to Tukey-Kramer test using software R (ver. 4.0.3).

Addition of skim milk, RNA, or DNA for extracting HMW DNA

To reduce the adsorption of HMW DNA onto soil components, 25 mg of skim milk, 10 mg of RNA from yeast (Roche, Switzerland), or 3 mg of sonicated salmon sperm DNA sodium salt (PanReac AppliChem, Germany) was added to 0.4 g of the soil sample SJ before adding the DNA extraction buffer. The subsequent procedures were similar to those of method I. As a sterile soil sample, a soil sample was autoclaved at 121°C for 60 min.

Addition of boiled salmon DNA

To reduce the contamination of soil sample HMW DNA with salmon DNA, the salmon sperm DNA sodium salt was suspended in TE buffer (100 mg ml^{-1}) and boiled for 15 min. Then, 30 µl of the solution was added to 0.4 g of the ground soil sample containing 1 ml of the DNA extraction buffer. To enhance the adsorption of salmon DNA onto the soil components, the mixture was incubated at room temperature (around 25°C) for 1 h before incubation with lysozyme at 45°C. The subsequent procedures were similar to those of method III. These procedures of method III with the boiled salmon DNA were termed as method IV in this study (Fig. 1).

References

Hoshino, T.Y., and Matsumoto, M. (2004) An improved DNA extraction method using skim milk from soils that strongly adsorb DNA. *Microbes Environ* **19**: 13-19.

Zhou, J., Bruns, M.A., and Tiedje, J.M. (1996) DNA recovery from soils of diverse composition. *Appl Environ Microbiol* **62**: 316–322.

Table S1 Properties of soil samples used in this study

soil sample name	origin	Soil type	total N g kg^{-1}	total C g kg^{-1}	pH (H_2O)
SA	paddy field, Ibaraki	Fluvisols	2.0	19.8	5.6
SB	paddy field, Fukuoka	Fluvisols	2.8	28.9	5.5
SC	spinach field, Kyoto	Fluvisols	1.5	14.8	7.2
SD	chingensai field, Kagawa	Regosols	0.5	8.5	7.5
SE	chingensai field, Kagawa	Regosols	2.0	32.0	7.6
SF	orchard, Kagawa	Regosols	0.8	9.2	5.0
SG	corn field, Ibaraki	Alisols	0.8	10.1	5.2
SH	corn field, Ibaraki	Gleysols	0.9	10.9	5.7
SI	corn field, Ibaraki	Andosols	2.1	29.3	5.6
SJ	corn field, Ibaraki	Andosols	3.0	45.8	5.3
SK	cabbage field, Ibaraki	Andosols	3.7	41.8	7.0

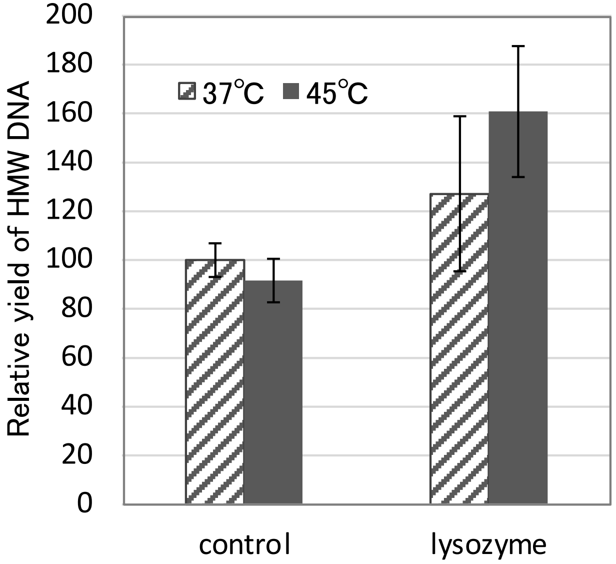

Figure S1 Effect of lysozyme on HMW DNA yield. 0.4 g of soil sample SA was grinded in liquid nitrogen and mixed with 1 ml extraction buffer. The soil suspension was incubated at 37℃ or 45℃ for 1 h with or without lysozyme under gentle inversion every 10 min. After proteinase K and SDS addition, the suspensions were incubated at 65℃ for 2 h under rotating vertically. The yield of collected crude DNA was evaluated by the signal intensity of the band in electrophoresis gel (n=3).

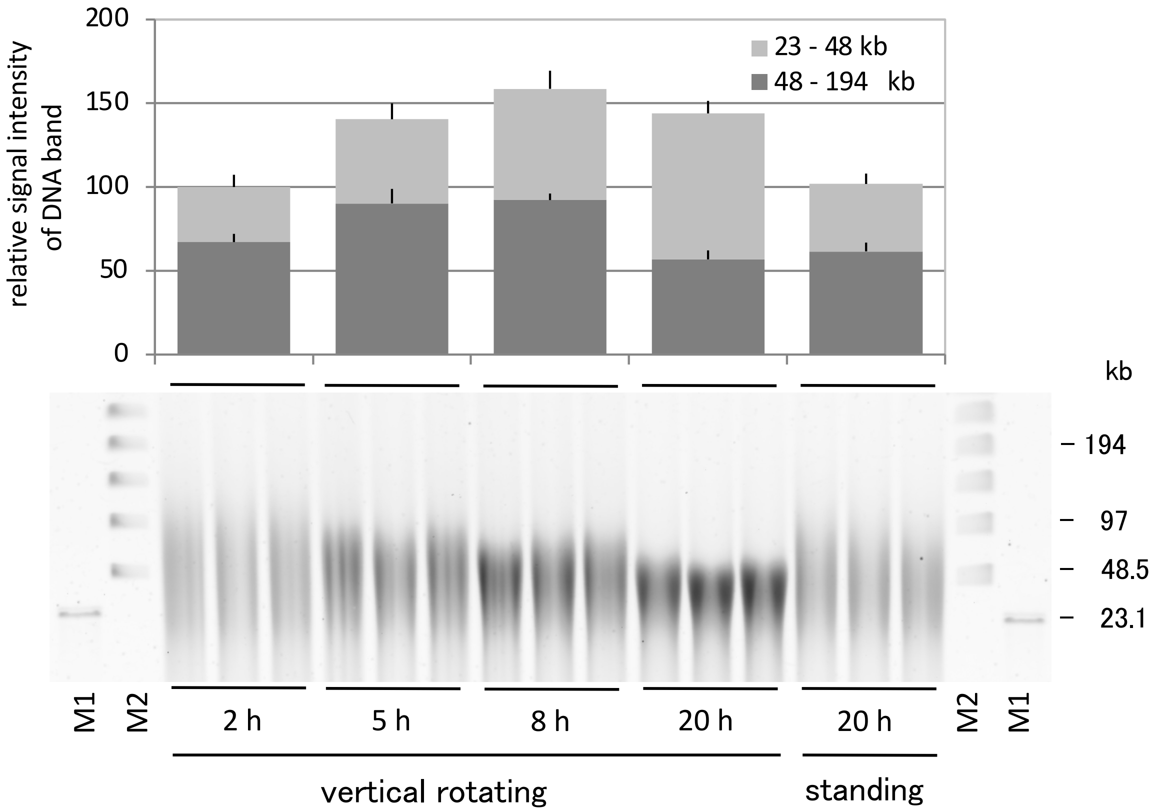

Figure S2 Effects of incubation time at 50°C with proteinase K and SDS on DNA length and yield. Image of PFGE gel (under part) and relative signal intensity of DNA band in the gel (upper part) are shown. Soil sample SA of 0.4 g was grinded in liquid nitrogen, mixed with 1 ml buffer and lysozyme, and incubated for 1 h at 45°C with vertical rotating. After adding protease and SDS, the soil suspensions were incubated at 50°C with vertical rotating for 2 to 20 hours or standing for 20 hours (n=3). M1: λ/HindIII; M2: lambda ladder

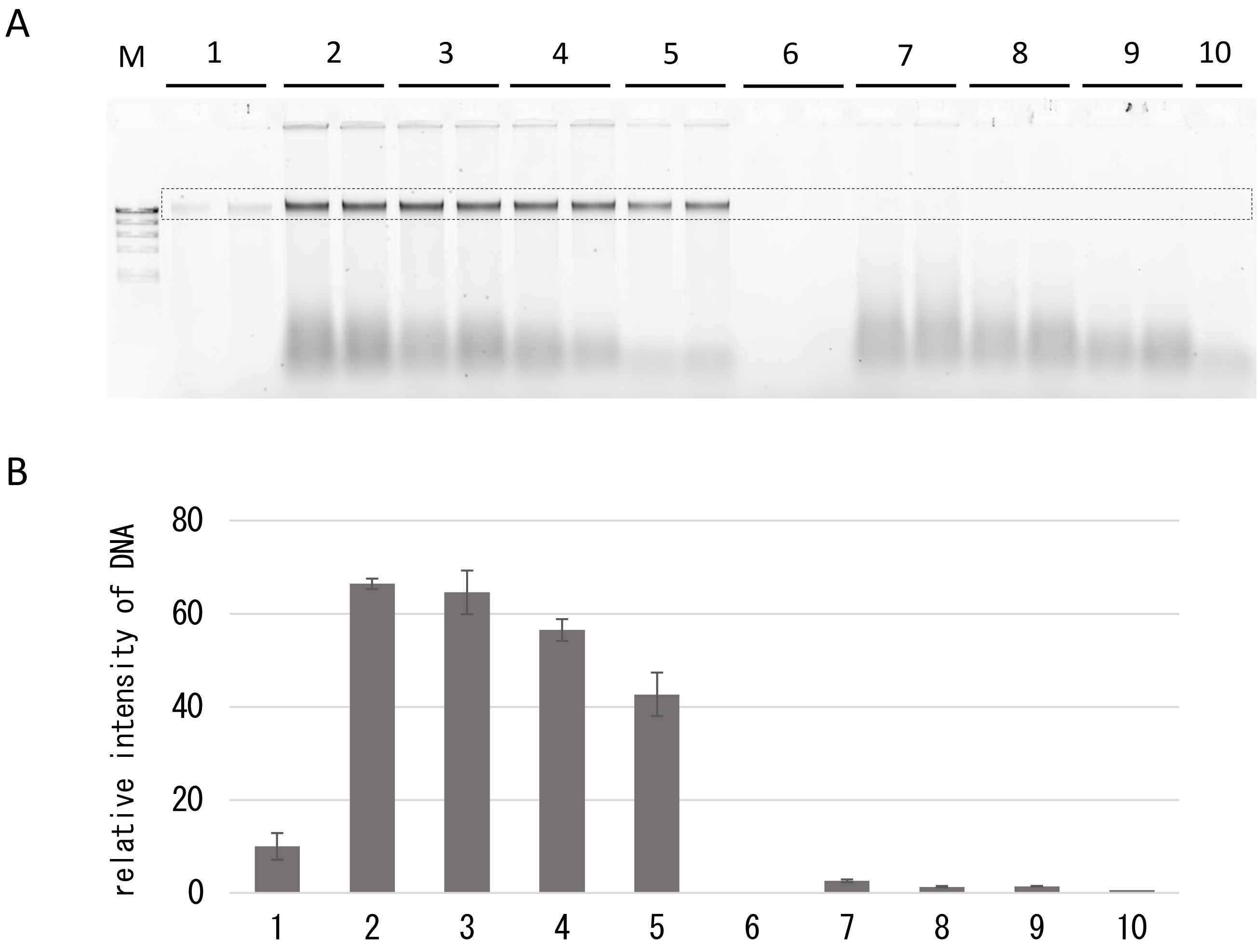

Figure S3 Effect of boiling or autoclaving treatment of salmon DNA on the extraction of HMW DNA from the soil sample SJ using method I. Electrophoresis-gel image of the crude HMW DNA (A) and relative intensity (B) of the the area surrounded by dotted line in the gel image. Lanes 1-5: non-sterile soil; 6-10: sterile soil. Lane 1and 6: no addition of salmon DNA; 2 and 7: non-boiled salmon DNA; 3 and 8: 15 min. boiled salmon DNA; 4 and 9: 30 min. boiled salmon DNA; 5 and 10: 1 min. autoclaved salmon DNA; M: λ/HindIII.

HMW DNA extraction from soil samples

1. Grind 0.4 g of soil sample under liquid nitrogen.

2. Transfer the grinded sample to 2 ml tube and add 1 ml of DNA extraction buffer (100 mM Tris-HCl [pH 8.0], 100 mM sodium EDTA [pH 8.0], 100 mM sodium phosphate [pH 8.0], 1.5 M NaCl, 1% CTAB).

   Option (reducing adsorption of HMW DNA onto soil particles); add 30 µl of boiled and shared salmon sperm DNA solution (100 mg ml^{-1} TE buffer) to the soil suspension. Stand for 1 h at room temperature.

3. Add 20 µl of lysozyme solution (500 mg ml^{-1} TE buffer) to the soil suspension and incubate for 1 h at 45 ℃ with vertical rotation.

4. Add 5 µl of proteinase K solution (20 mg ml^{-1} water) and 110 µl of 20% SDS to the soil suspension. Incubate at 50 ℃ for 5 h with vertical rotation.

5. Centrifuge at 20,000 *g* for 5 min at room temperature and collect the supernatant into 1.5 ml tube.

6. Mix the supernatant with equal volume of isopropanol, then incubate at room temperature for 1 h.

7. Centrifuge at 20,000 *g* for 15 min at room temperature. Wash the resulting pellet of crude DNA with 70% ethanol and resuspend the pellet in 100 µl of TE buffer.

Purification and size selection for cloning of long insert

(Scale-up of DNA extraction is recommended)

1. Purify the crude HMW DNA in supernatant of step 5 (left column) with phenol followed by chloroform: isoamyl alcohol (24:1). Precipitate and resuspend the DNA as step 6-7 (left column).

2. Separate the HMW DNA by size in pulse-field gel electrophoresis and cut out the gel block containing target size of the DNA.

3. Embed the gel block in low-melting-point agarose gel and transfer the HMW DNA into the agarose gel by electro-phoresis.

4. Cut out the gel block containing the HMW DNA and incubate the block with *β*-agarase.

5. Precipitate, wash and resuspend the HMW DNA as step 6-7 (left column).

Purification for PCR base analysis

1. Electrophoresis the crude HMW DNA of step 7 (left column) in 0.7% agarose gel and cut out gel brock containing HMW DNA.

2. Extract HMW DNA from the gel brock with a commercial kit (ex. QIAEX II gel extraction kit, QIAGEN).

Figure S4 Improved procedures for extracting HMW DNA from soil samples in this work, and examples of purification steps (Y. Sakai, unpublished).
